# Optical Screening of Novel Bacteria-specific Probes on *Ex Vivo* Human Lung Tissue by Confocal Laser Endomicroscopy

**DOI:** 10.3791/56284

**Published:** 2017-11-29

**Authors:** Bethany Mills, Ahsan R. Akram, Emma Scholefield, Mark Bradley, Kevin Dhaliwal

**Affiliations:** ^1^EPSRC Proteus Hub, MRC Centre of Inflammation Research, Queen's Medical Research Institute, University of Edinburgh; ^2^School of Chemistry, EaStChem, University of Edinburgh

**Keywords:** Immunology, Issue 129, Optical probe, bacterial infection, confocal laser endomicroscopy, distal lung, translation, intensive care unit, optical imaging.

## Abstract

Improving the speed and accuracy of bacterial detection is important for patient stratification and to ensure the appropriate use of antimicrobials. To achieve this goal, the development of diagnostic techniques to recognize bacterial presence in real-time at the point-of-care is required. Optical imaging for direct identification of bacteria within the host is an attractive approach. Several attempts at chemical probe design and validation have been investigated, however none have yet been successfully translated into the clinic. Here we describe a method for *ex vivo* validation of bacteria-specific probes for identification of bacteria within the distal lung, imaged by fibered confocal fluorescence microscopy (FCFM). Our model used *ex vivo* human lung tissue and a clinically approved confocal laser endomicroscopy (CLE) platform to screen novel bacteria-specific imaging compounds, closely mimicking imaging conditions expected to be encountered with patients. Therefore, screening compounds by this technique provides confidence of potential clinical tractability.

**Figure:**
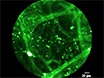


## Introduction

This technique describes a rapid screening process for evaluating bacteria-specific optical imaging agents within *ex vivo* human lung tissue by CLE using FCFM for the rapid identification of compounds with potential clinical utility for visualizing bacteria in the distal lung *in situ*.

There is an urgent global requirement to ration antimicrobial prescribing in the era of rising antimicrobial resistance^1^. To this end, the development of diagnostic methods which act to identify bacterial infection with high specificity, sensitivity, and in real-time are highly sought^2^. Current techniques to confirm a diagnosis of pneumonia in critically unwell patients, such as those within intensive care units (ICUs), often rely on interpreting non-specific clinical or radiological features alongside bacterial culture techniques from aspirated fluids/tissues, which can take up to 3 days to produce results. Furthermore, bacterial culture from fluid instilled into the distal lung and retrieved is prone to contamination from more proximal airways^3^ and is often culture negative due to concomitant antimicrobial therapy or poor sampling techniques. Additionally, molecular techniques such as polymerase chain reaction are overly sensitive when used on aspirated fluids, risking overtreatment of patients. An emerging diagnostic approach is molecular optical imaging, making *in situ *molecular pathology of tissues a possibility; however, the development and validation of optical imaging compounds is required. Nonetheless, direct visualization of bacteria, via activatable optical probes is potentially a very powerful method to allow the study of the presence and evolution of pneumonia in the patient, and importantly, could be used to study host-pathogen interactions in response to therapies in real-time *in situ. *

CLE is an established investigative procedure in multiple diseases^4^, including within the fields of gastroenterology^5^, oncology^6^,^7^, and for interrogating airways and alveolar sacs^8^,^9^. It enables point-of-care structural imaging of diseased tissue using a fiber imaging bundle, which passes through the working channel of a clinical endoscope and forms direct contact with the tissue surface to be imaged by confocal microscopy. However, one limitation that remains is the need for generic contrast agents. Therefore, the use of disease specific probes, such as specific bacterial agents, could vastly expand the utility of this modality by directly visualizing bacteria at the site of suspected infection. Optical agents offer many advantages over other techniques by enabling real-time, high resolution imaging with diagnostic potential. Moreover, optical probes offer the prospect of multiplexing for interrogating multiple targets, all achieved at a relatively low cost. A number of optical agents are under development for such a purpose, however none have yet been successfully implemented within the clinic^10^. We have synthesized a library of small molecule chemical probes with specificity towards bacteria and developed a rapid, effective pipeline for evaluation of probe function for detecting bacterial pneumonia *in situ*^11^.

To identify suitable probe candidates, the following prerequisites had to be fulfilled prior to interrogation of the probe on *ex vivo* human lung tissue by FCFM: i) aqueous solubility, ii) specificity and selectivity for rapidly labeling clinically relevant bacteria, iii) a high signal-to-noise ratio, and iv) resistance to degradation within the lung environment. The latter was assessed by bronchoalveolar lavage fluid (BALF) from patients with acute respiratory distress syndrome (ARDS), which is a condition that is characterized by proteolytic and inflammatory environments in the lung in the ICU. Moreover, the probes had to have a suitable fluorophore for detection by a clinically approved optical CLE imaging device within human lung alveolar tissue.

The pipeline to interrogate each of these prerequisites was as follows (at each stage, only probes that passed were carried forward to the next): (1) a library of probes to be investigated was synthesized; (2) each probe was added to a panel of live bacteria for confocal laser scanning microscopy (CLSM) to ensure bacterial labeling; (3) selectivity of bacterial labeling over mammalian cells in co-cultures with primary human neutrophils was established by CLSM; (4) stability and successful labeling of bacteria in the presence of the ARDS patient BALF was determined by CLSM and Matrix Assisted Laser Desorption/Ionization-Time of Flight (MALDI-TOF) Mass Spectrometry; (5) optimal concentration of candidates was determined by CLSM, ensuring selectivity for bacteria over mammalian cells was maintained; (6) candidates were imaged by FCFM in suspension and on *ex vivo* human lung alveolar tissue to ensure stability and that the signal-to-noise was adequate for detection. Step 6 is described in detail within this protocol. Methodology for steps 1 - 5 has been previously reported^11^.

## Protocol

All human lung tissue was obtained following informed consent and the study was approved by the Regional Ethics Committee.

### 1. Preparation of Biological Samples

1. **Preparation of probes**
  1. Make up a 1 mM stock solution of each probe (*e.g.*, Calcein AM, UBI-3, UBI-10, *etc.*) in sterile dH_2_O, using a fine balance to weigh the freeze-dried probe compound^11^. Calculate the volume of dH_2_O to add based on the weighed mass and molecular weight of the probe.
2. **Preparation of bacterial cultures** NOTE: For this method, *Staphylococcus aureus* was used as the exemplar strain. Any appropriate bacterial strain can be selected. Any commercial dye that labels bacteria with an appropriate excitation and emission spectra can be selected to positively label the bacteria.
  1. Select a single colony of desired bacterial strain from a fresh Lysogeny Broth (LB) agar plate using a sterile loop. Inoculate the colony into 10 mL of LB in a 50 mL centrifuge tube by dipping the end of the loop into the culture media. Incubate at 37 °C, 250 rpm for 16 h (or overnight).
  2. Determine the OD_595 _of the overnight culture by adding 100 µL of the overnight culture to 900 µL LB in a 1 mL cuvette. Measure the OD at 595 nm (using a cuvette with 1 mL LB as a blank) in a spectrophotometer. Multiply the obtained OD by 10 to obtain the OD for the overnight culture.
  3. Subculture the overnight culture. Do this by adjusting the optical density at 595 nm (OD_595_) to 0.1 in 10 mL of fresh LB. Calculate the required volume of overnight culture to be added to 10 mL fresh LB to adjust the OD_595_ to 0.1. Incubate the culture at 37 °C, 250 rpm until the culture reaches mid-log phase (OD_595_ 0.6 - 0.8), approximately 4 h.
  4. Measure the culture optical density (step 1.2.2) and harvest 1 x 10^8^ colony forming units (CFU) (OD_595_ ~1-1 x 10^8^ CFU/mL) of the bacterial culture into a 1.5 mL microtube (*e.g.*, if the bacterial culture is OD_595_ 0.6, collect 1.67 mL). Centrifuge the culture at 10,000 x g at room temperature for 1 min to pellet the bacteria. Wash the pellet twice in phosphate buffered saline (PBS) by resuspending (pipetting up and down carefully) the pellet in 1 mL PBS, centrifuging as above, discarding the supernatant, and repeating. Take care not to dislodge the bacterial pellet when removing the supernatant. Resuspend the final pellet in 1 mL PBS. NOTE: Prepare as many samples as required for each labeling procedure. The protocol may be paused here for up to 1 h, followed by step 1.2.5.1, 1.2.5.2, or 1.2.5.3.
  5. Bacterial staining
    1. To label the bacteria with Calcein AM, add the dye to a final concentration of 1 µM into the washed bacterial culture. Incubate the culture for 30 min at 37 °C with shaking at 300 rpm. Wash the counterstained bacterial suspension in PBS 3 times by centrifugation as in step 1.2.4 to remove excess dye. Resuspend in 1 mL PBS, dilute 100 µL 1:1 in PBS to obtain 200 µL of OD_595_ 0.5 Calcein AM stained bacteria. The protocol may be paused here for up to 1 h.
    2. To label the bacteria with test probes (*e.g.*, UBI-3 or UBI-10), dilute 100 µL of the bacterial culture 1:1 in PBS to obtain OD_595_ 0.5 in 200 µL PBS. Add either of the probes to a final concentration of 10 µM. Invert the microtube several times to ensure thorough mixing of the bacteria and the probe. NOTE: Imaging should be performed immediately after the addition of the probe to mimic the clinical scenario.
    3. To prepare the control unstained bacterial samples, dilute 100 µL of the culture 1:1 with PBS to obtain 200 µL of unstained OD_595_ 0.5 sample.
3. **Preparation of *ex vivo* human lung tissue** NOTE: Human lung tissue samples were obtained from patients undergoing surgical resection for lung carcinoma. All tissue used for imaging was obtained from samples of normal lung tissue away from the cancerous growth. Samples were taken fresh from the operating theatre and stored in microtubes or centrifuge tubes at -80 °C until use.
  1. Immediately before imaging, remove the human lung tissue sample from the freezer on dry ice. At room temperature, allow the tissue to thaw slightly; just enough to be sliced with a scalpel into 1 x 4 mm^2^ sections. NOTE: The level of thawing is important; too frozen and the tissue will not slice without chipping off, too thawed and the tissue is too soft to slice.
  2. Using forceps, place the sliced human lung tissue into wells of a 96-well clear flat-bottom tissue culture plate. Return any unused human lung tissue immediately to the storage container and place on dry ice for transport back to the -80 °C freezer.
  3. Add 100 µL PBS to each lung tissue sample with a pipette. Use the pipette tip to ensure all the tissue is covered in the PBS (and not stuck to the walls of the well). The tissue will swell slightly and may float. Leave the PBS on the sample for a few minutes to allow any blood to leach from the tissue into the solution. Remove as much of the PBS as possible. The tissue may block the end of the pipette tip; try to angle the plate/tip placement in order to prevent this.
  4. Pipette 100 µL of unstained, Calcein AM labeled, or test-probe labeled bacteria to each well containing lung tissue. Also set up controls with lung tissue and 100 µL PBS. To these control wells, probes without bacteria can be added to measure any increase in background fluorescence and/or non-specific activation of the probe by lung tissue alone. A well with lung tissue and PBS should also be included.

### 2. Imaging with the CLE Device with FCFM

1. **Set up of CLE device** NOTE: Prepare the CLE system 20 min before calibration to allow the laser to warm up.
  1. Press the on/off switch on the back of the transformer of the system and turn on the designated computer. Press the on/off button on the front of the laser scanning unit (LSU). Confirm the appearance of the green light, indicating that the unit is switched on.
  2. Double click on the CLE software icon. Enter the login details and wait for the LSU to initialize (10 - 30 s).
  3. For use of new FCFM imaging fibers, installation with the supplied CD is necessary. Insert the installation CD into the computer CD drive and follow the onscreen instructions.
  4. Clean the FCFM imaging fiber connector unit with a fiber cleaner. Rub the connector on the cleaning ribbon in a forward motion to remove any dust/dirt. Remove the protective yellow cap from the front of the LSU.
  5. Prepare the LSU hub by gently rotating the silver hub anti-clockwise until it stops. Insert the FCFM imaging fiber connector into the hub with the flat side of the connector facing upwards. Hold the fiber in place and rotate the silver hub clockwise until it clicks twice. Complete the connection by rotating the silver hub clockwise by a further 45°. NOTE: If the fiber is not recognized, check that the FCFM imaging fiber has been installed and connected in the correct orientation.
  6. Follow the instructions that will pop-up for completing the FCFM imaging fiber calibration. There are 3 steps: (1) FCFM imaging fiber test (steps 2.1.7 - 2.1.8), (2) background acquisition (step 2.1.9), (3) fiber detection (step 2.1.10).
  7. Press the 'start laser' button on the screen. The laser will center.
  8. Select fresh vials (yellow: calibrate; red: clean; blue: rinse) from the calibration kit and follow the onscreen instructions: place the distal end of the FCFM imaging fiber into the yellow vial and watch for the increase in fluorescence on the monitor, then place the fiber tip into the red vial (without stirring). Wait for the fluorescence (as shown on the computer monitor) to disappear. Finally rinse the fiber tip in the blue vial. NOTE: If the fluorescence does not disappear, clean the FCFM imaging fiber with 8% H_2_O_2 _and lens cleaning tissues and begin again. Repeat the process until satisfactory results are obtained (the image quality is clear, and no marks from dirt are apparent).
  9. Place the FCFM imaging fiber into the blue vial. Press 'start laser' followed by 'calculate' when this becomes an option.
  10. Place the FCFM imaging fiber into the yellow vial. Press 'start laser' followed by 'calculate' when this becomes an option.
  11. During the automated calibration, clean the distal end of the FCFM imaging fiber by placing in the red vial for >10 s, followed by the blue vial for >4 s, as indicated by the software.
2. **Data collection with CLE**
  1. Following setup, a window to select storage location and file prefix will open. Select the desired folder for data storage, and name the prefix accordingly.
  2. Place the foot pedals so that they can be easily accessed by the operator. Left pedal: laser on/off; center pedal: pause; right pedal: record/stop. NOTE: The laser controls can also be accessed through onscreen controls.
  3. Click 'start' onscreen or press the left foot-pedal to turn on the laser. This will start acquisition and obtain images using 100% laser power and a frame rate of 12 frames/s (default settings). NOTE: For other applications, these settings can be altered on screen if necessary, depending on the sample type.
  4. Image each of the bacterial suspension samples.
    1. Insert the distal end of the FCFM imaging fiber and move the fiber slowly through the suspension to interrogate the sample.
    2. Record videos of any length (up to 10 min) by pressing the right foot pedal or selecting the onscreen record controls, as the fiber moves slowly around the sample.
    3. Clean the distal end of the FCFM imaging fiber with 8% H_2_O_2 _and lens cleaning tissues between samples. ​NOTE: Typical video lengths of 10 - 30 s are sufficient for *in vitro *imaging.
  5. Image each of the lung tissue samples.
    1. Insert the distal end of the FCFM imaging fiber into the sample, ensuring that direct contact between the end of the fiber and the tissue is made. Gently move the end of the imaging fiber around to interrogate the sample. NOTE: Lifting the end of the fiber away from the tissue will remove the tissue from the focal plane; however, this may be used to image labeled bacteria that are not adhered to the tissue.
    2. Record videos of any length (up to 10 min) by pressing the right foot pedal or selecting the onscreen record controls, as the fiber moves slowly around the sample. NOTE: Typically, video lengths of 30 s are sufficient for *ex vivo* imaging on tissue.
    3. Clean the distal end of the FCFM imaging fiber with lens cleaning tissues and 8% H_2_O_2 _between samples.
3. **Turning off the system**
  1. Switch the laser off by pressing the left foot pedal or by clicking the on-screen button.
  2. Disconnect the FCFM imaging fiber from the CLE device by turning the silver LSU hub anticlockwise until it stops. Remove the FCFM imaging fiber from the LSU hub by gently pulling the fiber connector from the LSU.
  3. Clean and disinfect the FCFM imaging fiber with 8% H_2_O_2_ and lens cleaning tissues. Return the protective caps to the proximal end of the FCFM imaging fiber and the front of the LSU unit. Place the fiber gently in the storage box.
  4. Close the data capture software and copy any saved files to an external USB device. Shut down the computer and turn off the LSU device by pressing the front panel I/O for 3 s until the green light disappears.
  5. Dispose of human lung tissue and bacteria according to local regulations.

### 3. Data Analysis

1. Open the software and import the files for analysis by selecting the appropriate directory on the computer through the 'Go to' icon on the software dashboard. Alternatively, files can be dragged and dropped into the software dashboard.
2. Double click on each video file to open them. The videos will automatically play with optimized color lookup table (LUT) and color table adjust. Disable the automatic intensity scaling, by clicking on the wand button above the intensity scale bar. The feature is disabled when there is no black shadowing around the button. NOTE: Automatic intensity scaling must be disabled to prevent continuous contrast enhancement throughout each video, making it impossible to compare and analyze videos from the same data set.
3. Select the desired intensity scaling by moving the minimum and maximum bars to give the best contrast. Use the histogram tool when selecting the intensity scaling to ensure the broadest dynamic range is captured. NOTE: Ensure that the dynamic range is such that the images are not saturated (*i.e.* limit the white regions of the image, which indicate saturation), so that low intensity features are not missed.
4. Once the desired scaling has been achieved, right click over the dropdown menu button listed as 'Default (Green)'. Select the option to save the LUT. Save the LUT to a desired location.
5. For each other video within the data set, apply the same LUT by right clicking over the 'Default (Green)' drop down menu and select 'Load LUT'. Select the appropriate file to apply consistent intensity scaling to all videos within a dataset.
6. Export processed videos by clicking on the 'movie reel' button. Select the desired video format, *e.g. *'for presentation purposes', which will produce a .mpg file. Press 'Export' and chose file location to save the video file. Snapshots of single frames can be exported by clicking the 'camera' button. It is possible to save a .png, .bmp, or .jpg file. Choose the file destination and press save. NOTE: Videos can then be imported into any software for preparation of presentations or further quantification. Labeled bacteria are visualized as green 'blinking' dots in the video. The lung tissue structure will be apparent as ordered fluorescent strands, with alveolar space appearing black.

## Representative Results

In this study, we have demonstrated a method for the rapid-screening of novel bacteria-specific probes in an *ex vivo* human alveolar lung tissue model of infection using a clinically approved CLE device.

CLE by FCFM is well suited for obtaining structural information within the distal lung, as this region (due to a high abundance of elastin and collagen) is naturally highly fluorescent when excited with a 488 nm laser^8^. Conversely, the alveolar space does not fluoresce, and as such enables high contrast between tissue structure and air space to be visualized (Figure 1).

The addition of disease related probes or contrast agents, such as bacteria-specific probes should enable functional information about disease processes to be obtained in real-time. We have previously described the synthesis and initial *in vitro* screening of a library of bacteria-specific probes^11^; where bacterial-specificity, proteolytic stability, and retention within the bacterial membrane over time was determined. A promising bacteria-specific probe (UBI-10) was identified within the study, as well as one that showed poor retention within the bacterial cell membrane (UBI-3). These were compared to a control of commercial counterstaining (Calcein AM) that was used to labeled *S. aureus*.

Unstained, Calcein AM, UBI-3, and UBI-10 labeled *S. aureus* were imaged in suspension by FCFM with 100% 488 nm laser power and a frame rate of 12 frames/s (Figure 2). Where unlabeled bacteria in PBS were imaged, no fluorescent signal was detectable. This is in contrast to when labeled bacteria were imaged. Where bacterial suspensions with UBI-3 or UBI-10 were imaged by FCFM, it was apparent that the general background fluorescence of the solution was elevated compared to PBS only controls, this is because NBD (the probe fluorophore) does emit a small amount of fluorescence signal in aqueous solution, however, bright punctate dots are clearly visible throughout the solution, without the need for a wash step. This is due to an increase in fluorescence signal emitted from NDB in a polar environment *i.e.*, the bacterial membrane. Calcein AM is not an activatable probe, so a wash step after bacterial staining was required to remove the high fluorescent background of unbound probe in the solution. Like UBI-3 and UBI-10 labeled bacteria, bacteria labeled with Calcein AM were detected in solution by FCFM as bright green punctate dots. As the data are collected in video format, these dots appear to 'twinkle' as they move between cores and in-and-out of focus, a characteristic trait of imaging labeled bacteria by this method.

The labeled bacteria were subsequently added to small slices of *ex vivo* human lung tissue and imaged again by FCFM (Figure 3). Where only PBS or unlabeled *S. aureus* was added to the lung tissue, only the lung tissue autofluorescent structure was detected (seen as bright green strands of collagen and elastin and dark areas of alveolar space). No punctate dots were detected for these control conditions. Similarly, only lung tissue structure (and no punctate dots) was visualized for the lung tissue condition with *S. aureus* plus UBI-3; indicating that this probe was not retained stably within the bacterial cell membrane *i.e.*, it was washed out and/or is degraded in the presence of native proteolytic enzymes within the lung tissue (as previously demonstrated^11^).

However, bright punctate dots were visible in both the Calcein AM labeled positive control *S. aureus *sample, and with the most promising bacteria-specific probe (UBI-10) *S. aureus *sample. The 'twinkling' dots were visible despite the strong tissue autofluorescence (Figure 3). Thus, the results obtained by FCFM were in concurrence with the *in vitro* pre-screening of the panel of bacteria-specific probes by CLSM, and demonstrated a clinically relevant detection method for imaging infections in real-time.

The results presented here demonstrate that the lung is an appropriate organ system for imaging by FCFM due to its distinctive autofluorescence. The bright distinctive structures allow the CLE operator to determine that they are in the alveolar space. These regions, coupled with the dark alveolar air sacs provide the perfect backdrop for imaging fluorescently labeled bacteria with high contrast.

Although the detection of bacteria presented within this study is determined qualitatively by visualizing bright punctate dots, it could be possible to quantify the number of punctate dots frame by frame using a secondary software in order to further characterize probe libraries.

**Figure:**
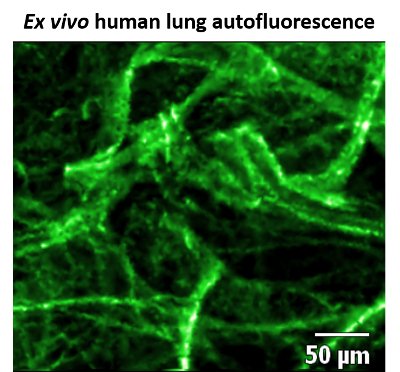

Figure 1:** Static image of human lung tissue autofluorescence.** Confocal laser endomicroscopy (CLE) image of *ex vivo* human lung tissue using fibered confocal fluorescence microscopy (FCFM), at 488 nm excitation, 100% laser power, and 12 frames/s. Elastin and collagen are highly fluorescent (false colored green), whereas alveolar space is not, and appears as black regions. This figure has been modified from Akram *et al*.^11^ Please click here to view a larger version of this figure.

**Figure:**
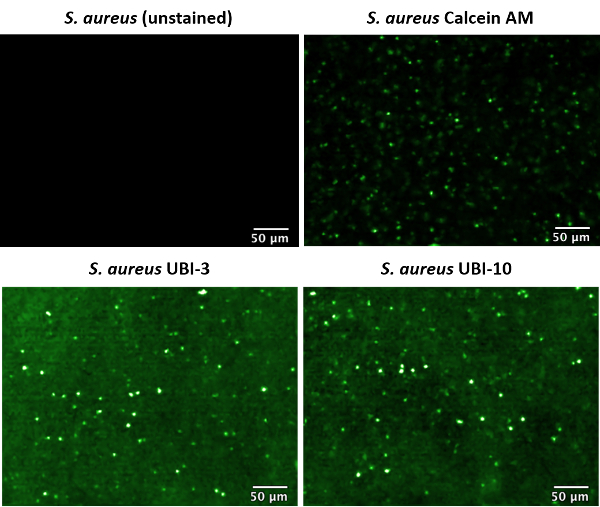

Figure 2:** Confocal laser endomicroscopy (CLE) image of labeled* S. aureus* in suspension. **Fibered confocal fluorescence microscopy (FCFM) was used to image pre-labeled bacteria, at 488 nm excitation, 100% laser power, and 12 frames/s. Labeled bacteria show as highly fluorescent punctate dots (false colored green). This figure has been modified from Akram *et al*.^11^ Please click here to view a larger version of this figure.

**Figure:**
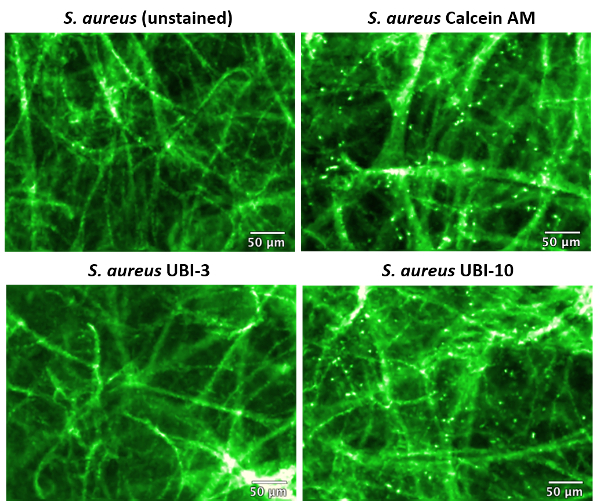

Figure 3:** Confocal laser endomicroscopy (CLE) image of* ex vivo* human lung tissue with labeled* S. aureus*.** Fibered confocal fluorescence microscopy (FCFM) was used to image *ex vivo *human lung tissue and labeled *S. aureus*, at 488 nm excitation, 100% laser power, and 12 frames/s. Labeled bacteria show as highly fluorescent punctate dots (false colored green) within the lung tissue sample when labeled with Calcein AM or UBI-10. The highest contrast is observed where bacteria are imaged within the alveolar space. This figure has been modified from Akram *et al*.^11^  Please click here to view a larger version of this figure.

## Discussion

Lower respiratory tract infections account for the second highest burden of disease globally^12^,^13^, and a substantial rise in the number of infections attributed to antimicrobial resistant bacteria has been reported^14^. Pneumonia remains a common cause for hospitalization. In the ICU, the development of a pneumonia is compounded by diagnostic uncertainty and is associated with an extremely high mortality rate^15^. During the onset of pneumonia, bacteria proliferate within the alveolar space of the distal lung, an area that is relatively sterile, with minimal microbiota in health.

This method describes relatively late stage *ex vivo* validation of bacteria-specific optical imaging probes^11^, but the design, synthesis, and probe evaluation prior to beginning this validation step is imperative, as previously shown^11^.

CLE is an emerging clinical technique for interrogating disease states *in situ* in real-time. It offers many advantages over traditional techniques for investigating suspected pulmonary pathology, which may involve a biopsy and collection of the lavage fluid. Biopsies are invasive and can cause morbidity and mortality in ventilated patients, and collected lavage fluid is often contaminated with bacteria from the upper airways. The use of CLE in the detection of pneumonia is however somewhat limited due to the poor availability of compatible imaging probes which may provide functional information of disease, despite many concerted efforts^10^. Combining CLE with optical agents offers the prospect of diagnosing pneumonia faster and less invasively compared to current standard practice.

The critical steps to this protocol are in the sample preparation and setup of the CLE platform. Obtaining human tissue relevant to the final clinical application is also important, such as human lung tissue as demonstrated within this study. It is necessary to use human tissue because the extent of tissue autofluorescence shows large inter-species variation, and may therefore mislead the sensitivity of the bacteria-probe being imaged. Additionally, obtaining ethics for retrieval and use of human lung tissue is essential. From a technical level, correct cleaning, attachment of the imaging fiber to the imaging LSU platform, and calibration is essential for good resolution and consistent imaging, as is ensuring equivalent numbers of bacteria are added to each lung tissue sample. To further expand the utility of this method for screening panels of probes, repeating the procedure with a range of pathogens, such as those likely to be causative agents of pneumonia is necessary.

The largest limitation of this technique is that the clinically approved CLE device has only one laser (488 nm). Therefore, currently, the selection of fluorophore for probe design is limited for use with this system, though clinically approved single color devices do exist with excitation wavelengths of 660 nm and near-infrared. It is highly desirable to have a second laser line implemented within the same device to enable a probe to be developed with a spectrally distinct fluorophore to improve bacteria-probe sensitivity over the level of tissue autofluorescence. Whilst dual-color CLE devices are under development, they are either not clinically approved and/or their cost is significant^16^.

CLE *in vitro* using pathogenic bacteria and *ex vivo* human lung tissue to screen potential probes bridges the gap between conventional *in vitro *techniques such as flow cytometry and CLSM, and clinical utility. This step offers confidence when selecting promising compounds to carry forward to be coupled with clinical CLE imaging; and will provide indication as to whether the tested probe maintains target specificity, or demonstrates any off-target labeling, such as binding directly to tissue, or shows instability with host proteolytic enzymes. It would also be pertinent to add each of the activatable probes directly to samples of human lung tissue plus bacteria, to fully characterize the speed of probe binding and activation in real-time.

We believe that our pipeline for rapidly screening novel bacteria-specific probes to assess their potential for imaging within the distal lung of patients will result in much faster translation to the clinic. This is largely because the bacteria-specific probe could be delivered locally within the lung through a catheter inserted down the working channel of a bronchoscope, meaning that microdose (<100 µg) amounts could be delivered. Therefore, systemic delivery and biodistribution of the compound is not a concern, as is the case for many other infection targets within the body, or with nuclear imaging. Moreover, delivering the imaging probe in such a small dose reduces the risk of toxicity related complications (although toxicity screening would be required for translation). Following instillation of the probe, the catheter could then be replaced by the FCFM fiber and the same region of the lung interrogated by CLE, much the same way we have performed within this method. Imaging should be performed rapidly following installation of the probe before the probe washes away to undetectable concentrations.

It is also important to note that screening of disease-identifying probes by this technique should not be limited to bacterial-imaging agents, but could also extend to the validation of probes with alternative targets, such as inflammation. This approach should also be adaptable to other disease locations within the body where imaging via FCFM is permissible.

## Disclosures

KD: Founder Director of Edinburgh Molecular Imaging. Received consultancy from Mauna Kea Technologies as advisor. 

MB: Founder Director of Edinburgh Molecular Imaging. 
